# Transcription Factors Associated With IL-15 Cytokine Signaling During NK Cell Development

**DOI:** 10.3389/fimmu.2021.610789

**Published:** 2021-03-18

**Authors:** Xiang Wang, Xiang-Yu Zhao

**Affiliations:** ^1^ Peking University People’s Hospital, Peking University Institute of Hematology, Beijing Key Laboratory of Hematopoietic Stem Cell Transplantation, National Clinical Research Center for Hematologic Disease, Beijing, China; ^2^ Beijing Engineering Laboratory for Cellular Therapy, Beijing, China

**Keywords:** IL-15, signaling/signaling pathways, natural killer cell, development, transcription factor

## Abstract

Natural killer (NK) cells are lymphocytes primarily involved in innate immunity and possess important functional properties in anti-viral and anti-tumor responses; thus, these cells have broad potential for clinical utilization. NK cells originate from hematopoietic stem cells (HSCs) through the following two independent and continuous processes: early commitment from HSCs to IL-15-responsive NK cell progenitors (NKPs) and subsequent differentiation into mature NK cells in response to IL-15. IL-15 is the most important cytokine for NK cell development, is produced by both hematopoietic and nonhematopoietic cells, and functions through a distinct delivery process termed transpresentation. Upon being transpresented to NK cells, IL-15 contributes to NK cell development *via* the activation of several downstream signaling pathways, including the Ras–MEK–MAPK, JAK–STAT5, and PI3K–ATK–mTOR pathways. Nonetheless, the exact role of IL-15 in NK cell development has not been discussed in a consecutive and comprehensive manner. Here, we review current knowledge about the indispensable role of IL-15 in NK cell development and address which cells produce IL-15 to support NK cell development and when IL-15 exerts its function during multiple developmental stages. Specifically, we highlight how IL-15 supports NK cell development by elucidating the distinct transpresentation of IL-15 to NK cells and revealing the downstream target of IL-15 signaling during NK cell development.

## Introduction

NK cells constitute the third most abundant lineage of lymphocytes in the peripheral blood after B and T cells, accounting for approximately 8–15% of circulating cells in humans or 2–6% in mice (1). Similar to CD8^+^ cytotoxic T lymphocytes, NK cells effectively eliminate virus-infected cells and malignant cells by producing proinflammatory cytokines and directly lysing target cells. NK cell activation is determined by the balance between signals transduced from multiple activating receptors and inhibitory receptors, which interact with their cognate ligand on target cells (2, 3).

Interleukin (IL)-15, a member of the common gamma chain cytokine family, was first described as a T cell growth factor, like IL-2 (4). IL-15 signals through a heterotrimeric receptor consisting of IL-15R*α* (CD215), IL-2/IL-15R*β* (CD122) and the common *γ* chain (*γ*c, CD132) (5). Similar to IL-2, IL-15 requires the receptors IL-2/IL-15R*β* and *γ*c to transduce signaling but differs from IL-2 by virtue of its private binding receptor IL-15R*α*, which is incapable of transducing signaling but has high affinity for IL-15 and forms a complex (IL-15–IL-15R*α*) in IL-15-expressing cells (4, 6, 7). These IL-15/IL-15R*α* complexes have the potential to stimulate neighboring cells that express IL-2/IL-15R*β* and *γ*c *via* a unique mechanism referred to as tanspresentation (8, 9). Since the discovery of transpresentation, increasing evidence has suggested that IL-15 responses are largely mediated by transpresentation at steady state (10, 11).

NK cells primarily develop in the bone marrow (BM), which contains abundant hematopoietic stem cells (HSCs) capable of differentiating toward NK cells through common lymphoid progenitor (CLP) and lineage-restricted progenitor (NKP) cells (12). Multiple internal pathways and external factors contribute to the development of NK cells from HSCs (13). Most importantly, the pleiotropic cytokine IL-15 is indispensable for the development and homeostasis of NK cells as highlighted by their significant deficiency in IL-15-deficient mice. Correspondingly, deficiency in IL-15 or any one of the IL-15 receptor subunits, such as the IL-15R*α*, IL-15R*β*, and *γ*c in mice, results in a dramatic paucity of mature NK cells (14–17). Parallel with the role of IL-15 in mice, several studies have demonstrated that the early commitment of NK cells from human CD34^+^ hemopoietic progenitor cells into NKP cells is dependent on the coordinated function of IL-3, IL-7, c-kit ligand (KL), and flt3 ligand (FL) but not IL-15, whereas IL-15 is involved in the emergence of CD56^+^ NK cells (18, 19). Furthermore, Huntington et al. demonstrated that human NK cell differentiation that occurs in a linear fashion from CD56^hi^CD16^−^KIR^−^ to CD56^lo^CD16^+^KIR^−^ and finally to CD56^lo^CD16^+^KIR^+^ requires IL-15 in a humanized model (20). Collectively, these findings illustrate that IL-15 signaling is essential for NK cell development and homeostasis in both mice and humans. Interestingly, Sun et al. recently reported that the requirement of IL-15 for NK cell development could be partially overcome by acute mouse cytomegalovirus (MCMV) infection, as IL-12 but not IL-15 primarily drives the anti-viral response of NK cells even in mice lacking the *γ*c (21). Further studies are required to investigate whether this represents an IL-15-independent NK cell development manner.

Due to the critical role of IL-15 in NK cell development, dissecting the signaling pathways that allow IL-15 to control the development and homeostasis of NK cells is fundamental to determine the molecular details of immune regulation. In this review, we provide an overview of the specific IL-15 signaling that transcriptionally regulates NK cell development and maturation.

## When Does IL-15 Promote NK Cell Development?

### IL-15 Is Dispensable for NK Cell Commitment but Promotes Later Development

Mice and human NK cells are generated from HSCs through multiple but sophisticated stages in specific developmental niches with internal and external regulatory pathways governing NK cell development. In brief, NK cell development primarily involves the following two independent and continuous processes: early NK cell commitment to IL-15-responsive NKPs and subsequent phenotypical and functional maturation of NK cells in response to IL-15. Early NK cell commitment to NKP cells is characterized by the acquisition of CD122 (IL-15R*β*), which is a critical subunit of the IL-15 receptor and dimerizes with *γ*c to transduce IL-15 signaling (22, 23). However, IL-15 is not involved in the generation of IL-15-responsive NKPs because the IL-15 receptor is not expressed prior to the NKP stage (24). Recently, pre-NKP cells were identified as the earliest committed NK cell progenitors in murine BM, and these cells reside downstream of CLP and differentiate into NKPs (23). Although pre-NKP cells express undetectable levels of CD122, they are fully committed to the NK lineage both *in vitro* and *in vivo*. Therefore, IL-15 is not necessary for NK cell lineage commitment. Furthermore, mice deficient in *γ*c exhibit an intact NKP compartment (16), and IL-3, IL-7, KL, and FL synergistically drive the differentiation of NKP cells from human HSCs *in vitro* in the absence of IL-15 (25). Conversely, IL-15 is indispensable for the later development of NK cells. The expression of CD122 endows NK cells with the capacity to be responsive to IL-15; thus, these cells can become phenotypically and functionally mature and exhibit survival in response to IL-15 (16).

### IL-15 Receptor Expression Varies in Different Stages of NK Cell Development

Intriguingly, the expression of CD122 on NK cells is not static but dynamically changes with NK cell maturation. It has been previously demonstrated that CD56^bright^ NK cells express higher levels of CD122 as well as elevated CD122 transcripts compared with CD56^dim^ NK cells, and thus are intrinsically more responsive to IL-15 (26–29). This observation explains the decreased proliferation capacity in response to IL-15 or dendritic cell (DC) stimulation during NK cell maturation (30, 31) and is consistent with the fact that cytokines, such as IL-2 and IL-15, fail to reverse the proliferation defects of CD57^+^ terminally matured NK cells (32). Consistent with the observation in human NK cells, CD122 expression is significantly decreased during maturation from mice CD11b^+^CD27^+^ NK cells to CD11b^+^CD27^−^ NK cells and concomitant with decreased proliferation capacity (33). Despite the vitally important role of IL-15 in NK cell maturation, the exact role of decreased CD122 expression during NK cell terminal maturation needs to be further elucidated.

### Transcriptional Regulation of IL-15 Receptor Expression at Different Stages During NK Cell Development

Although CD122 (encoded by Il2rb) is critical for NK cell development by transducing IL-15 signaling, the coordinated regulation of CD122 expression by various transcription factors remains elusive. Previous studies have demonstrated that RUNX3 (one of the Runx family transcription factors), T-bet, and Eomesodermin (Eomes) directly bind to the promoter region of Il2rb and induce CD122 expression (34, 35). However, these transcription factors are not simultaneously functional, but rather function at different stages of NK cell development. In the NK cell development pathway, RUNX3 expression is initiated at the NKP stage. The inactivation of RUNX3 in HSCs partially disturbed the generation of CD122^+^NKP cells *in vitro* but not completely, indicating that other unknown transcription factors contribute to the expression of CD122 during NK cell commitment. In addition, the deletion of RUNX3 in immature NK cells in mice only slightly reduced CD122 expression on NK cells, and the absolute number of NK cells was not significantly affected. These results confirmed that RUNX3 is necessary for the acquisition of CD122 during NK cell lineage commitment but is not essential for the maintenance of CD122 at the later maturation stages of NK cell development (34).

In contrast, T-bet and Eomes are weakly expressed at the NKp stage but highly expressed during NK cell maturation; therefore we speculated that T-bet and Eomes are not firmly involved in the induction of CD122 at the NKp stage but may contribute to the maintenance of CD122 expression during maturation (35, 36). Consistently, mice harboring genomic deletions of T-bet and Eomes lack NK cells, but CD122^hi^ precursors of NK cells were observed (36). In addition, the deletion of Eomes in mice results in significantly decreased CD122 expression at different stages of NK cell maturation (33, 35, 37). Moreover, Eomes^+^ NK cells express more CD122 and proliferate better than Eomes^−^ NK cells, which are called Innate Lymphoid Cells (ILCs) 1 now (38, 39). However, CD122 expression is upregulated in T-bet-deficient NK cells, and this finding may be attributed to increased Eomes expression, which is repressed by T-bet (40). These results indicate that Eomes but not T-bet plays a dominant role in the maintenance of CD122 expression during NK cell maturation. Consistently, although T-bet expression is upregulated during the NK cell transition from the CD11b^+^CD27^+^ to CD11b^+^CD27^−^ stage, CD122 expression is progressively decreased, accompanied by a reduction in Eomes expression (33).

**Figure 1:**
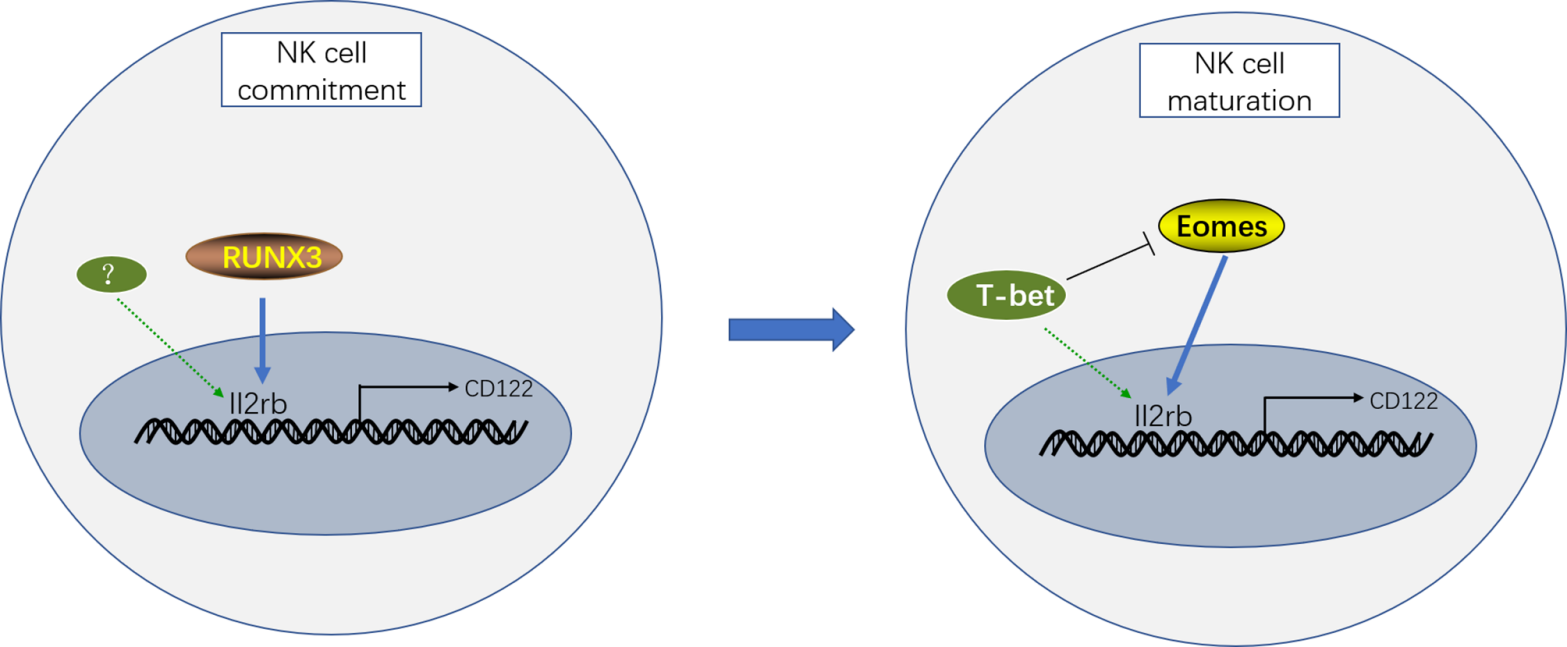
Transcriptional regulation of CD122 expression during different stages of NK cell development. RUNX3 coordinates with T-bet and Eomes to control CD122 expression during NK cell development. Therein, the induction of CD122 expression during early NK cell commitment is determined by RUNX3 and other unknown transcription factors. Moreover, both T-bet and Eomes contribute to CD122 expression during NK cell maturation. However, Eomes but not T-bet plays a predominant role in the maintenance of CD122 expression to promote NK cell maturation.

In conclusion, the induction of CD122 during early NK cell commitment is dependent on RUNX3, whereas Eomes but not T-bet maintains the expression of CD122 to promote NK cell maturation (**Figure 1**).

## Which Cells Produce IL-15 to Promote NK Cell Development?

### Isolated Expression of IL-15 mRNA and Protein

IL-15 mRNA is constitutively expressed in a broad range of tissues, including hematopoietic cells [monocytes, macrophages, and dendritic cells (DCs)] and non-hematopoietic cells (epithelial cells, fibroblasts, nerve cells, skeletal muscle and keratinocytes) (4). In contrast to the widespread expression of IL-15 mRNA, IL-15 protein is only detectable in a more restricted population at steady state. This discrepancy between widespread IL-15 transcript expression and restricted protein expression is attributed to extensive checkpoints at transcription, translation, and intracellular trafficking, particularly post-transcriptional checkpoints. Multiple 5′-untranslated region (UTR) AUG sequences, a long signal peptide (LSP) (48 amino acid) and a negative regulatory element in the C-terminus of the coding sequence and mature protein all contribute to impede translation (41–44). Surprisingly, a 250-fold increase in IL-15 expression is observed after the removal of those three predominant restraints, further demonstrating the contribution of multiple post-transcriptional mechanisms in limiting IL-15 translation (42). Additionally, there are two isoforms of IL-15 mRNA, differing in their signal peptide, which result in distinct intracellular trafficking, localization, and secretion patterns (44–46). Both isoforms produce mature IL-15 protein. IL-15 with LSP is primarily located in the Golgi apparatus, early endosomes, and endoplasmic reticulum and functions as a secretory signal peptide, whereas IL-15 with a short signal peptide (SSP) (21 amino acid) is not secreted, appears to reside in the nucleus and cytoplasmic components (44). Tight regulation of IL-15 expression is important because of the potent capacity of IL-15 to promote inflammation.

### The Production of IL-15 by Hematopoietic and Non-Hematopoietic Cells

Due to the extremely low level of IL-15 protein expression at steady state, even after stimulation, IL-15 is barely detectable by antibodies. However, the establishment of an IL-15 reporter mouse line allows IL-15-producing cells to be visualized by flow cytometry or fluorescence microscopy as well as immunohistochemistry *in vivo* (47–49). Among hematopoietic cells, IL-15 is predominantly produced by monocytes, macrophages, DCs, myeloid cells, and some early hematopoietic cells (**Table 1**) (4, 47). Therein, CD8^+^conventional DCs are the major DC subsets responsible for IL-15 expression rather than plasmacytoid DCs (47, 48). Moreover, myeloid cells, including neutrophils, basophils, and eosinophils, express high levels of IL-15 *in vivo*, whereas lymphoid lineages, such as T cells, B cells, NKT cells, and NK cells, express minimal to undetectable IL-15 levels. Interestingly, LSK cells (Lineage^−^Sca-1^+^c-kit^+^), which constitute a heterogeneous population of both long-term and short-term HSCs in BM, uniformly express high levels of IL-15.

**Table 1:** The production of IL-15 by hematopoietic cells and non-hematopoietic cells.

Hematopoietic cells	Non-hematopoietic cells
Monocytes Macrophages DCs: CD8^+^conventional DCs Myeloid cells: neutrophils, basophils and eosinophils Early hematopoietic cells: LSK cells	In the BM: CXCL12-abundant reticular (CAR) cells In the thymus: thymic medulla and medullary thymic epithelial cells with high MHC class II expression In the lymph nodes: fibroblastic reticular cells (FRCs), gp38^−^CD31^−^ stromal cells and blood endothelial cells (BECs) In the spleen: VCAM-1^+^ stromal cells

Among nonhematopoietic cells, a distinct category of stromal cells together with epithelial cells directs IL-15 expression in primary and secondary lymphoid organs (**Table 1**) (49). In BM, IL-15 is predominantly expressed by VCAM1^+^PDGFRβ^+^CD31^−^Sca-1^−^ mesenchymal stromal cells, which correspond to a distinct subset of CXC chemokine ligand-12 (CXCL12)-abundant reticular (CAR) cells and may function as a developmental niche for NK cells (50, 51). In the thymus, IL-15 is highly expressed in the thymic medulla and medullary thymic epithelial cells with high MHC class II expression, providing a major source of IL-15. In the lymph nodes, IL-15-expressing cells, which include some fibroblastic reticular cells (FRCs) and gp38^−^CD31^−^ stromal cells, primarily reside in the T-cell zone and medulla. In addition, in the lymph nodes, blood endothelial cells (BECs) also express high IL-15 levels. In the spleen, VCAM-1^+^ stromal cells are responsible for IL-15 expression.

In contrast to the low expression of IL-15 at steady state, its expression capacity is further strengthened by several inflammatory stimuli, including Toll-like receptor (TLR) ligands and cytokines (47, 52–54). Previously, studies have proven that bacterial lipopolysaccharide (LPS) or the double-stranded RNA mimic Poly I:C initiates TLR signaling to induce IL-15 expression (55). Similarly, IL-15 induction is interferon (IFN)-*α* receptor (IFNAR)-dependent after viral infection (47). Although IL-15 mRNA is elevated in all DC subsets after inflammatory stimuli, only CD8*α*+ DCs upregulated IL-15 protein expression, further specifying a DC subset for IL-15 production (47, 55, 56). Moreover, it has been demonstrated that upregulated IL-15 expression also exists in monocytes, macrophages, and tumor-associated neutrophils in inflammatory environments (57–59). In addition, LPS-induced inflammation also greatly increases IL-15 expression in stromal cells, including BECs and lymphatic endothelial cells (LECs), whereas this effect is not significant in other stromal cells (49).

### Both Hematopoietic and Non-Hematopoietic Cells Promote NK Cell Development by Producing IL-15

**Figure 2:**
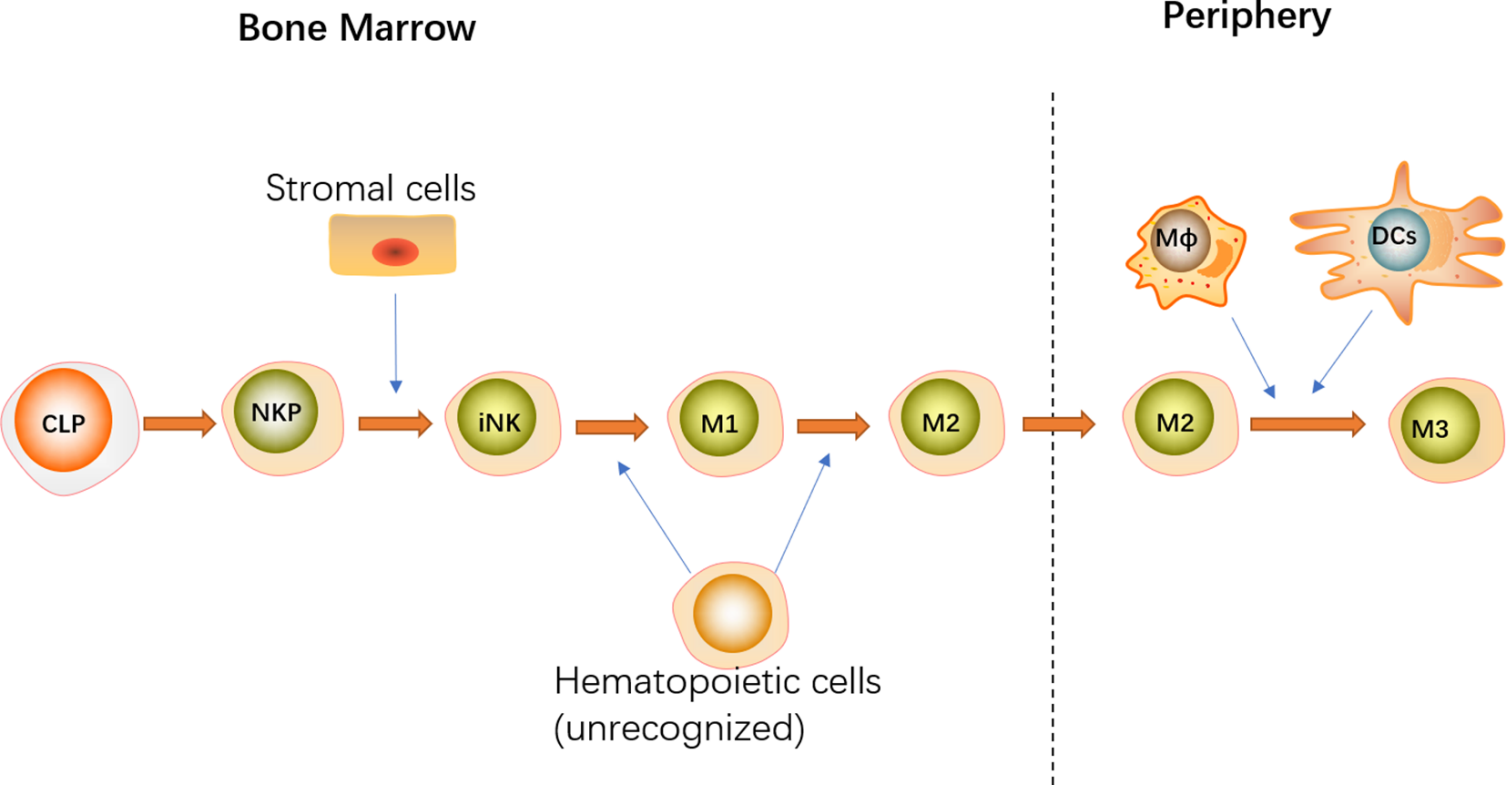
NK cell commitment is represented by the transition from CLPs to NKPs that acquire the most representative hallmark CD122. With the expression of NK1.1 and NKp46, immature NK (iNK) cells are originated from NKPs. According to the expression of CD27 and CD11b, NK cell maturation can be distinguished into four stages: CD11b^−^CD27^−^(iNK) → CD11b^−^CD27^+^(M1) → CD11b^+^CD27^+^(M2) → CD11b^+^CD27^−^(M3). Stromal cells expressing IL-15α are sufficient for the generation of immature NK cells in the BM. Moreover, some unrecognized hematopoietic cells contribute to differentiate into M1 and M2 NK cells by supplying IL-15. After migration from BM to the periphery, DCs and macrophages transpresent IL-15 for M2 to promote NK cell terminal maturation. Both hematopoietic and non-hematopoietic cells promote NK cell development by producing IL-15 at different stages.

The diverse subsets of IL-15-expressing cells play different but overlapping roles in the development of NK cells in BM and peripherally by producing and transpresenting IL-15 (**Figure 2**). Overall, hematopoietic cells were found to override the importance of non-hematopoietic cells in promoting NK cell development (10, 11). Correspondingly, restricting IL-15R*α* or IL-15 expression to hematopoietic cells completely recovered NK cell development at all stages in BM with a slight defect in peripheral mature NK cells, whereas the development of NK cells was only partially rescued in all tissues when IL-15R*α* or IL-15 expression was specifically limited to non-hematopoietic cells.

As a critical component of hematopoietic cells, monocytes, DCs, and macrophages contribute to NK cell development by producing IL-15. The indispensable role of monocytes in NK cell development and homeostasis was exemplified by the observation that the interaction between NK cells and spleen monocytes promotes CD11b^+^CD27^+^ NK cell differentiation into CD11b^+^CD27^−^ NK cell in an IL-15R*α*- and IL-15-dependent and cell–cell contact-dependent manner (60). Consistently, immunobiological studies revealed that monocytes and NK cells reside in close proximity of the red pulp of the spleen (60). Additionally, although IL-15R*α* expression on DCs and macrophages is dispensable for NK cell differentiation in BM, it is required for the maintenance of mature NK cells in the periphery given that specific knockdown of IL-15R*α* on DCs or macrophages results in a substantial reduction in NK cells in the periphery (61). Moreover, NK cell homeostasis is not exacerbated when IL-15R*α* is conditionally deleted from both DCs and macrophages, indicating that DCs and macrophages maintain NK cell populations in the peripheral blood and organs in a similar manner (61). Furthermore, mice with conditional deletion of IL-15R*α* in DCs or macrophages exhibit significant deficits in terminally differentiated CD27^−^CD11b^+^ NK cells, although the subsets of peripheral CD27^+^CD11b^−^ and CD27^+^CD11b^+^ NK cells remain intact. Thus, DCs and macrophages were dispensable for NK cell development in the BM and necessary for NK cells’ terminal differentiation in the periphery. However, using CD11c/IL-15R*α* Tg mice with an IL-15R*α* ^−/−^ background, Castillo et al. revealed that DCs contribute to the development of NK cells in both the BM and peripheral blood and organs. Mice that exclusively expressed IL-15R*α* on DCs exhibited partial recovery of NK cells in all tissues, and the greatest reconstitution was noted in the BM (10). Furthermore, IL-15 exclusive transpresentation *via* DCs is insufficient for the maturation of CD27^−^CD11b^+^ NK cells, which preferentially reside in the peripheral blood and organs. These discrepancies in the function of DCs during NK cell development may be attributed to divergent models. Nonetheless, transpresentation of IL-15 by DCs and macrophages is not responsible for the all IL-15 events attributed to IL-15R*α*+ hematopoietic cells, as NK cell deficiency in the BM of mice with IL-15R*α* deletion in DCs or macrophages is less apparent than that observed in IL-15R*α*-deficient mice (10, 61). Therefore, besides DCs and macrophages, other unrecognized hematopoietic cells in the BM that contribute to NK cell development have not been identified.

Moreover, IL-15 expression by non-hematopoietic cells is more important for NK cell development in BM other than in the periphery, as limiting IL-15R*α* expression to non-hematopoietic cells results in more evident NK cell recovery in BM, and this effect is virtually non-existent in the spleen or liver (10). Non-hematopoietic cells expressing IL-15*α* are sufficient for the generation of immature NK cells but are incapable of NK cell maturation (10). This finding may be attributed to the high expression of IL-15 in CXCL12-abundant reticular (CAR) cells, which are in close contact with NK cells in BM (49–51). In addition to transducing the downstream signaling of CXC chemokine receptor (CXCR4), the engagement of CXCL-12 on CAR cells *via* CXCR4 expressed by NK cells also contributes to NK cell retention in BM, which provides a special IL-15-sufficient niche for NK cell development. *In vivo* and *in vitro* studies demonstrated that the CXCL-12/CXCR4 axis is essential for NK cell maturation and proliferation (50, 51). However, the exact role of IL-15 expression in CAR cells is unidentified. Additionally, consistent with the high expression of IL-15 in fibroblastic reticular cells (FRCs) of lymphoid nodes, the specific ablation of IL-15 in FRCs results in almost complete abrogation of NK cells in Peyer’s patches (PPs) and gut-associated secondary lymphoid organs (SLOs), indicating that FRCs promote NK cell homeostasis *via* the establishment of an IL-15-dependent niche (62).

Furthermore, human spleen-derived fibroblasts are sufficient for the development of functional CD56^bright^CD3^−^ NK cells *in vitro*, and neutralizing IL-15 signaling or disturbing direct contact significantly abrogates CD56^dim^CD3^−^ NK cell generation, indicating that fibroblasts express and transpresent IL-15 to support NK cell development (63). However, no *in vivo* studies have demonstrated the role of fibroblasts in NK cell development. In conclusion, although previous studies have uncovered the distinct function of DCs and macrophages in NK cell development, the exact biological role of IL-15 expression in other hematopoietic cells (myeloid cells and early HSCs) and diverse stromal cells that reside in the BM or peripheral blood and organs during NK cell development remains poorly described.

## How Does IL-15 Transpresentation Support NK Cell Development?

**Figure 3:**
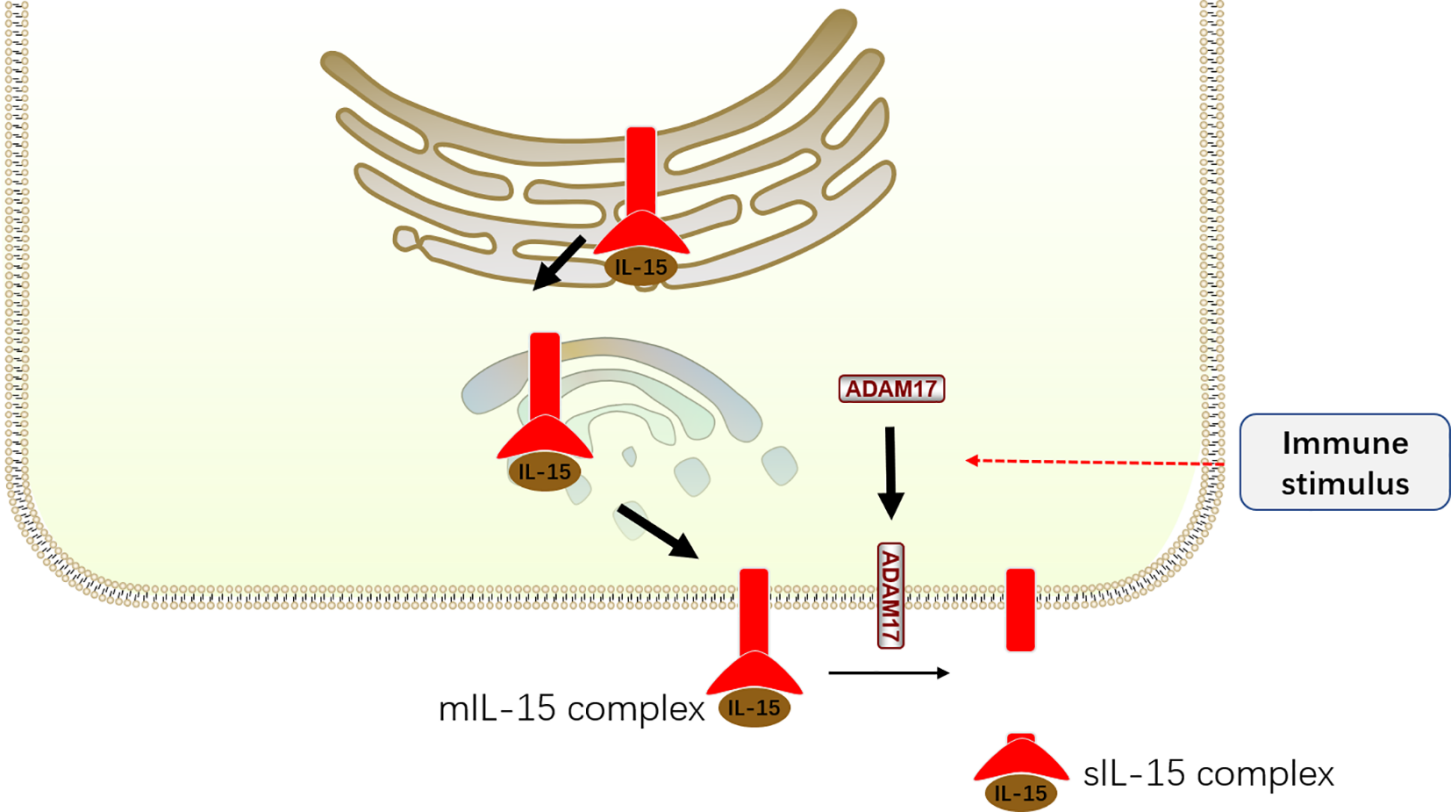
The production of mIL-15 complex and sIL-15 complex. IL-15 preassembles with IL-15R*α* in a complex in the endoplasmic reticulum/Golgi and subsequently shuttled to the cell surface, becoming membrane-associated IL-15–IL-15R*α* complex (mIL-15 complex). After immune stimulating, the ADAM17 translocates to the cell surface with increased activity. The mIL-15 is cleaved by ADAM17 at the ectodomain of IL-15R*α* to induce the formation of soluble IL-15–IL-15R*α* complex (sIL-15 complex).

Although IL-15 is critical for NK cell development, IL-15 alone only weakly activates its downstream signaling. In fact, the exertion of IL-15 function is dependent on IL-15R*α*, which has high affinity to IL-15 (64). With the aid of IL-15R*α*, IL-15 is protected from degradation, accumulates on the membrane and in the circulation of mice, and exhibits increased biological activity (65). Accordingly, IL-15-expressing cells must simultaneously express IL-15R*α* to supply IL-15 to IL-15-responsive NK cells bearing IL-15R*β* and *γ*c (66, 67). The distinct requirement is further unveiled by the discovery that IL-15 is preassembled with IL-15R*α* in a complex in the endoplasmic reticulum/Golgi and subsequently shuttled to the cell surface (8, 68). This cell surface complex is called membrane-associated IL-15-IL-15R*α* complex (mIL-15 complex) (**Figure 3**).

Nonetheless, the mIL-15 complex could be cleaved from the surface to form soluble IL-15–IL-15R*α* complex (sIL-15 complex) in response to several immune stimuli, including type I interferons (type I IFNs), Poly I:C stimulation, total body irradiation (TBI), Toll-like receptor (TLR) stimulation, virus infections, and activation of the stimulator of IFN genes (STING) pathway (57, 69, 70). It is reported that this process is mediated by A Disintegrin and Metalloprotease (ADAM) 17 protease, whose expression is upregulated on the surface of IL-15 expressing cells after immune stimulus (69). Consistently, *in vivo* evidence demonstrated that the IL-15–IL-15R*α* complex exists in two forms, mIL-15 complex and sIL-15 complex, in humans and mice (65, 70). Although the sIL-15 complex was identified several years ago, its biological significance remains controversial. Interestingly, *in vivo* studies revealed that the sIL-15 complex serves as a potent agonist and is approximately 50–100 times more potent at promoting NK cell proliferation than recombinant IL-15 alone (71, 72). Thus, the sIL-15 complex may play an important role in stimulating IL-15 responses. Consistently, Anton et al. (73) demonstrated that low doses of sIL-15 contribute to the phosphorylation of Stat5 in NK cells, whereas higher concentrations of sIL-15 are required to stimulate S6 phosphorylation *in vitro*. However, other studies discovered that the mIL-15 complex mediates NK cell activation rather than the sIL-15 complex present in the supernatants of IL-15-expressing cells cultured *in vitro* or in the serum of mice *ex vivo (* 68, 74 *).* This contradiction may be attributed to the different experimental methods and low concentration of sIL-15 complex in the supernatants and serum. Considering the rare detection of sIL-15 complex at steady states and substantial sIL-15 complex produced after immune stimulation, we hypothesize that the sIL-15 complex mediates IL-15 responses during immune activation but not during steady states. However, due to the technological limitation, it is hard to distinguish IL-15 responses mediated by sIL-15 complex from mIL-15 complex.

In contrast to the sIL-15 complex, which functions independently of cell–cell interactions, the mIL-15 complex functions through a distinct delivery mechanism termed transpresentation during cell–cell contact to transduce IL-15 signaling to NK cells *via* the IL-12/IL-15Rβ and *γ*c complex (8, 64). Consistently, although IL-15R*α* knockout mice exhibit dramatic defects in NK cell development (15), the specific deletion of IL-15R*α* in NK cells has no detrimental effect on NK cell development. However, adoptive transfer of normal NK cells into IL-15R*α*-deficient mice results in the abrupt loss of these cells, indicating that IL-15R*α* expressed by non-NK cells but not NK cells is required to mediate IL-15 signaling for NK cell development (75, 76).

**Figure 4:**
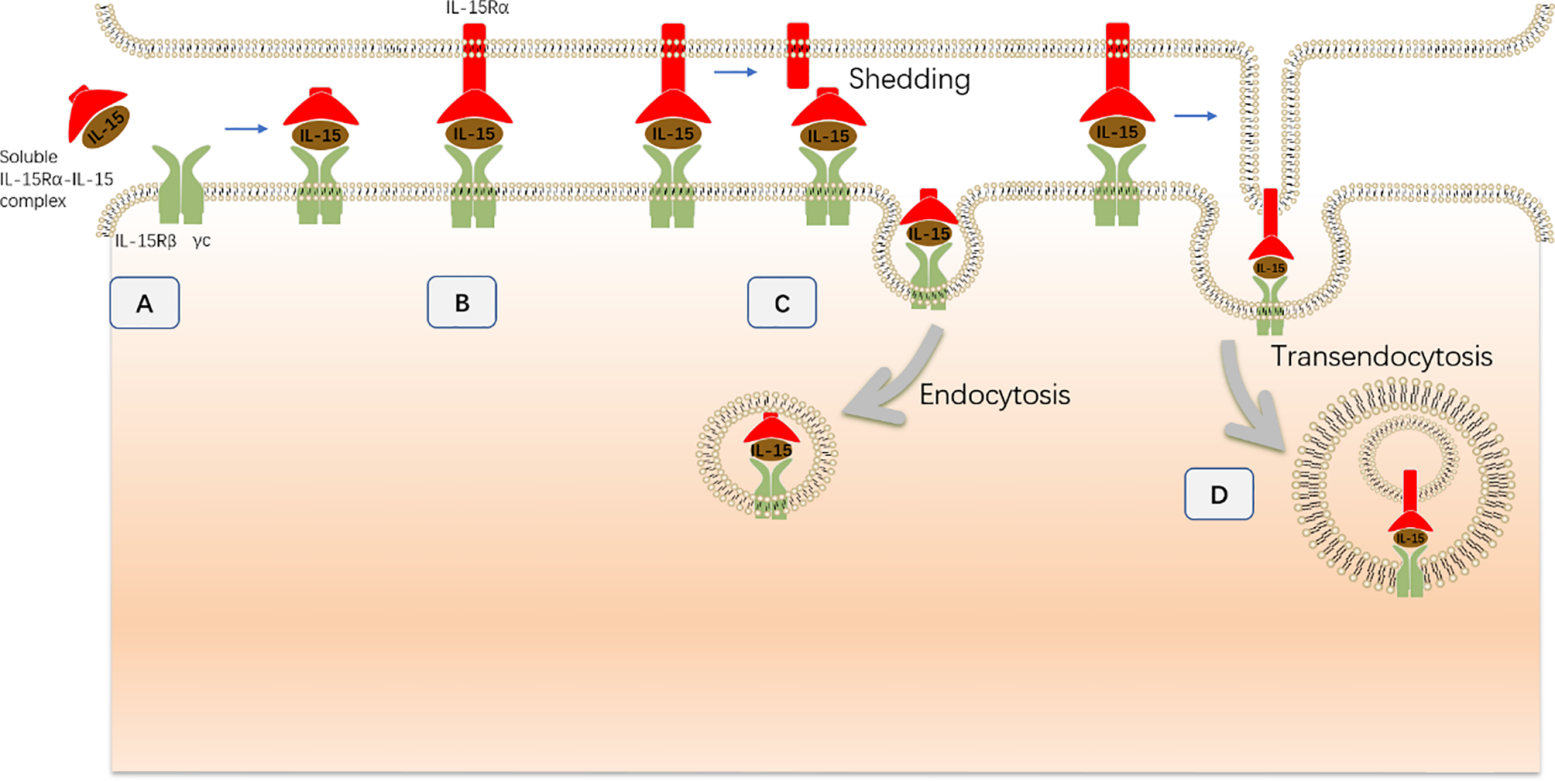
The transpresentation of IL-15 to NK cells during NK cell development. **(A)** The sIL-15 complex (soluble IL-15–IL-15R*α* complex), which exists as a soluble extracellular complex in serum, directly interacts with NK cells that express IL-15R*β*–*γ*c chains without transpresentation by presenting cells. **(B)** Presenting cells directly interact with NK cells *via* the formation of an immunologic synapse. Then, the mIL-15 complex (membrane-associated IL-15–IL-15R*α* complex) on presenting cells interacts with the IL-15R*β*–*γ*c receptor at the plasma membrane of NK cells to transduce IL-15 signaling. **(C)** During synapse formation, the mIL-15 complex is cleaved from the plasma membrane and then endocytosed by NK cells. The internalized mIL-15 complex co-localizes with the IL-15R*β*–*γ*c receptor in the cytoplasm and contributes to IL-15 signaling activation. **(D)** The intact mIL-15 complex together with the plasma membrane of presenting cells is internalized into NK cells through a distinct process termed transendocytosis without being cleaved. Subsequently, the transendocytosed IL-15R*α*–IL-15 complex colocalizes with the IL-15R*β*/*γ*c chain in intracellular NK cell compartments and contributes to IL-15 signaling activation.

During transpresentation, the IL-15R*α*–IL-15 complex functions through three different mechanisms to transduce IL-15 signaling in NK cells (73, 77) (**Figure 4**). First, presenting cells can directly interact with NK cells *via* the formation of an immunologic synapse where the membrane-associated IL-15R*α*–IL-15 complex on presenting cells interacts with the IL-15R*β*-*γ*c receptor at the plasma membrane of NK cells to transduce IL-15 signaling. Consistently, the mIL-15 complex expressed by DCs accumulates at the synapse with NK cells, and the use of an antibody to block IL-15R*α* promotes NK cell apoptosis and significantly reduces NK cell survival (78). In addition to the IL-15/IL-15R*α*-*β*/*γ*c interaction, many other receptor–ligand interactions may simultaneously occur at NK cell immunologic synapses, such as interactions between activating receptors or inhibitory receptors and their ligands, separately (78, 79). Interestingly, using a confocal microscopy assay, the mIL-15 complex accumulated in the periphery of activating synapses, whereas the mIL-15 complex was evenly distributed along the entire contact area when the NK cell line made contact with IL-15-expressing cells (79). Nonetheless, the regulatory role of these receptor–ligand interactions in IL-15 signaling remains elusive. *In vitro* studies have demonstrated that the interaction between inhibitory receptors, such as KIR2DL1, KIR2DL2/3, or CD94-NKG2A, and their cognate ligands selectively inhibited the phosphorylation of AKT and S6 but not Stat5, and this effect was concomitant with reduced proliferation induced by the mIL-15 complex but not the sIL-15 complex (79).

During cell-to-cell contact, the membrane-bound IL-15R*α*–IL-15 complex is internalized by NK cells and contributes to the activation of IL-15 signaling (77). This process is dependent on the proteolytic cleavage of IL-15R*α*, which allows the IL-15R*α*–IL-15 complex to separate from the presenting cells. In addition, the IL-15R*α*–IL-15 complex gradually accumulates in NK cells during the interaction between IL-15-presenting cells and NK cells. After separation from the presenting cells, the previously restored IL-15 complex contributes to the survival and residual proliferation of NK cells in a time-limited manner. In contrast, abrogation of IL-15R*α* cleavage results in enhanced and prolonged Stat5 phosphorylation concomitant with increased IL-15 expression in the synapse. This observation further demonstrated that the mIL-15 complex on presenting cells also contributes to the activation of IL-15 signaling during cell-to-cell contact (77). Therefore, mIL-15 complex cleavage and internalization could represent a negative regulatory mechanism that reduces the availability of transpresented-IL-15 and protects NK cell from excessive IL-15 signaling.

However, inhibition of IL-15R*α* cleavage did not completely abrogate the entry of the IL-15R*α*–IL-15 complex into NK cells, indicating that the IL-15 entry is not exclusively dependent on the shedding of the membrane-associated IL-15R*α*–IL-15 complex (77). Indeed, the intact membrane-associated IL-15R*α*–IL-15 complex from the presenting cells together with the plasma membrane of presenting cells is internalized into NK cells through a distinct process termed transendocytosis without being cleaved (73). Subsequently, the transendocytosed IL-15R*α*–IL-15 complex colocalizes with the IL-15R*β*/*γ*c chain in intracellular NK cell compartments to promote ribosomal protein S6 phosphorylation and NK cell proliferation. Consistently, interference of transendocytosis by silencing the small GTPase TC21, which is a critical component of transendocytosis, substantially inhibits S6 phosphorylation but not Stat5 phosphorylation in NK cells.

## What is the Downstream Target of IL-15 Signaling During NK Cell Development?

### IL-15-JAK-STAT5 Signaling for NK Cell Development

**Figure 5:**
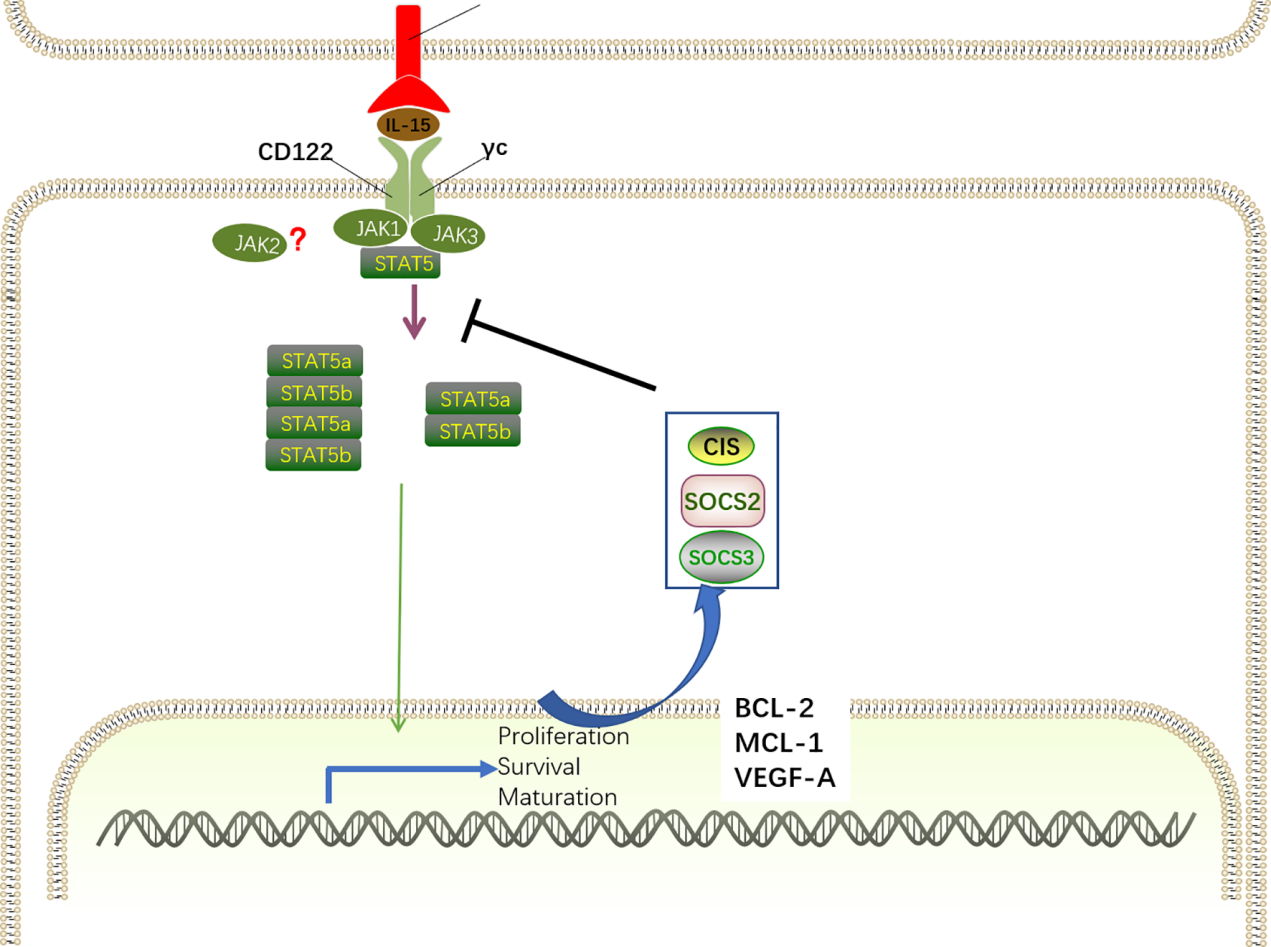
IL-15-JAK-STAT5 signaling for NK cell development. The IL-15R*α*–IL-15 complex primarily induces the activation of the JAK–STAT5 pathway *via* recruiting JAK1 and JAK3. For signal transduction, JAK1 binds to the IL-2/IL-15R*β* and JAK3 combines with *γ*c, inducing the recruitment and phosphorylation of STAT5. By oligomerizing into dimers and tetramers, phosphorylated STAT5a and STAT5b translocate into the nucleus to drive the expression of STAT-target genes encoding proteins related with NK cell development, survival, proliferation, and function, including MCL-1, BCL-2, and VEGF-A. Specifically, IL-15–JAK–STAT5 signaling also promotes the transcription of SOCS family members. SOCS proteins comprise a negative feedback loop to retrain the JAK–STAT5-mediated pathway in NK cell development.

Upon the engagement of the IL-15R*α*–IL-15 complex with the IL-15R*β*/*γ*c receptor, three distinct signaling pathways, including Ras–MEK–MAPK, JAK–STAT5 and PI3K–ATK–mTOR are activated and contribute to NK cell development. The IL-15R*α*–IL-15 complex primarily induces the activation of the JAK–STAT5 pathway *via* recruiting Janus kinase 1 (JAK1) and JAK3 (**Figure 5**). Interestingly, JAK1 binding to the IL-2/IL-15R*β* and JAK3 combining with *γ*c is crucial for signal transduction by activating JAK1 and JAK3, which induce the phosphorylation of tyrosine residues in IL-2/IL-15 R*β* (80–82). This model has been further confirmed by the discovery that humans with deletion of JAK3 exhibited similar phenotypes of severe combined immunodeficiency (SCID) as *γ*c-deficient patients (83). Although the specific function of JAK1 and JAK3 varies considerably, genetically engineered mice provide the possibility to determine the distinct roles of individual proteins. While deficiency of Jak1 in mice leads to perinatal lethality (84), a remarkable decrease of immature B220+ NK cells was observed in adult mice with inducible loss of Jak1, indicating that Jak1 is essential for NK cell development (85). These observations were recently validated in mice with conditional deletion of Jak1 in Ncr1-expressing cells (Jak1^fl/fl^ Ncr1Cre), displaying blockade of NK cell development at the NKp and iNK stages in a dose-dependent manner (86). Not surprisingly, JAK3 also plays an important role in NK cell development, coinciding with the finding that Jak3-deficient mice suffer from differentiation block of NK cells at the pre-NKP stage (87). Despite the cooperation of Jak1 and Jak3 in NK cell development, accumulating evidence has proposed that Jak1 plays a dominant role overriding Jak3 during the signal transduction (88, 89). The specific inactivity of Jak3 in human cells lines fails to attenuate STAT5 phosphorylation as anticipated, as two Jak kinases have an equivalent function in signal transduction, whereas remarkable abrogated downstream signaling was found in Jak1-inactive cells lines (88). Furthermore, the knockdown experiments suggested that Jak1 is responsible for the phosphorylation of Jak3 and STAT5 after cytokine receptor activation, and Jak3 contributes to enhance Jak1 activity by phosphorylating it. Likewise, quantitative mass spectrometry analysis also revealed that Jak1 is more important that Jak3 in mature NK cells (90). Although, many studies have addressed the vital roles of Jak1 and Jak3 in signal transduction, molecular interactions between the two Jak kinases and their individual contributions in NK cells remain to be determined.

Although it has long been believed that IL-15 signaling is exclusively mediated by JAK1/3, the role of JAK2 in IL-15 signaling is controversial. Notably, a recent study described that JAK2 phosphorylates STAT5 downstream of IL-15 during NK cell differentiation *in vitro* (91). Accordingly, mice with conditional deletion of Jak2 in HSC exhibited impaired NK cell maturation (92). However, it has been shown that JAK2 is intrinsically dispensable for NK cell development as mice with conditional deletion of JAK2 in NKp46+ cells exhibited intact NK cell numbers and maturation (86). These discrepancies indicate that the absence of JAK2 may extrinsically interfere in NK cell maturation by altering the cytokine milieu.

The discovery of the IL-15-JAK association has contributed to the finding that members of the signal transducer and activation of transcription (STAT) family directly bind to phosphor-tyrosine docking site(s) in the IL-2/IL-15R*β* chain and are then phosphorylated by JAK1 and JAK3 on their tyrosine residues (80). Similar with IL-2, IL-15 predominantly induces STAT5 activation, despite the finding that STAT3 and STAT1 can also be activated to a lesser extent (93). STAT5 is comprised by two distinct transcription factors, STAT5a and STAT5b, that have a remarkable degree of sequence homology (approximately 96%) (94). By oligomerizing into dimers and tetramers, phosphorylated STAT5a and STAT5b translocate into the nucleus to drive the expression of STAT-target genes, which is critical for NK cell development, survival, proliferation, and function (95–97). STAT5 dimers preferentially bind to *γ*-interferon-activated sequence (GAS) motifs, whereas STAT5 tetramers are more flexible given the capacity for various non-consensus GAS motifs (98). Interestingly, Lin and colleagues revealed that STAT5 dimers are sufficient for early NK cell development, proliferation and cytotoxic capacity, whereas STAT5 tetramers are necessary for NK cell maturation and survival through the induction of the anti-apoptotic protein Bcl2 (99).

It is indisputable that STAT5-related transcriptional programs mediated by IL-15 activation are essential for the biological functions of IL-15. The indispensable role of STAT5-mediated transcriptional regulation in NK cell development has been highlighted by the finding that NK cell differentiation was abrogated at the NKp stage in Ncr1-iCreTg mice with conditionally deleted STAT5 (100). Consistently, disrupted NK cell maturation and impaired lytic function were observed in humans with STAT5b mutations (101). Therefore, STAT5 downstream of Jak kinases is essential for transducing IL-15 signaling. Despite the largely redundant functions of STAT5a and STAT5b, their distinct roles have been verified in single knockout mice for STAT5a or STAT5b (102, 103). Deficiency of STAT5b results in more dramatic defects in NK cell development than deletion of STAT5a, indicating that STAT5b plays a dominant role in NK cell development (104, 105). Consistently, transcriptional analysis revealed that the transcripts mediated by STAT5b are more abundant (104). Furthermore, only Stat5b knockout mice exhibit elevated transcription of VEGFA, an angiogenic factor that is transcriptionally repressed by STAT5.

Moreover, chromatin immunoprecipitation (ChIP) analysis of STAT5 binding sites revealed that STAT5 directly targets a large number of genes encoding proteins related with NK cell development and function, including ID2, EOMES, T-BET, perforin, granzymes, and IFN-*γ*. Additionally, STAT5 can also bind to Mcl-1 and Bcl-2, correlating with the ability of IL-15 to induce the expression of these genes and sustain NK cell survival (97, 106). Overexpression of BCL-2 enables the survival of STAT5-deficient NK cells but has no influence on proliferation, maturation, or effector functions. However, it seems that Mcl1 is more important in promoting NK cell survival than Bcl-2, as IL-15 stimulation maintains NK cell survival when Bcl-2 was inhibited but not when Mcl1 was inactivated (96).

However, STAT5 is not only correlated with transcriptional activation of gene expression, as the repressive effect of STAT5 binding is also present in NK cells. STAT5 has been shown to bind the Vegf-a gene promoter in NK cells, correlating with suppressed expression of the pro-angiogenic factor VEGF-A in mice and humans (106). *In vitro* studies revealed that STAT5-inactive NK cells showed abundant VEGFA expression, and this effect was also confirmed *in vivo* by increased tumor formation in the absence of STAT5 (106). In line with the observations in mice, tumor-infiltrating NK cells with VEFGA secretion properties promote tumor progression and are associated with poor outcomes in patients (107–109). According to the repressive effects of STAT5 on IL-17 and Bcl6 mRNA expression in T cells (110, 111), further research is essential to deepen our understanding of the distinct roles of STAT5 in NK cells.

IL-15 signaling contributes to the induction of suppressor of cytokine signaling (SOCS) family members, including cytokine inducible SH2-containing protein (CIS), SOCS2, and SOCS3, which comprise a negative feedback loop to retrain the IL-15–JAK–STAT5-mediated pathway in NK cell development (90, 112). Several studies have demonstrated that STAT5 directly targets the genes of these SOCS proteins (99, 113). SH2-containing protein (CIS, encoded by Cish gene) directly interacts with JAK1 to mediate the inhibition of its enzymatic activity and proteasomal degradation, thereby constraining JAK–STAT5 signaling. Consistently, mice with CIS ablation exhibit accumulation of terminally differentiated CD27^−^CD11b^+^ NK cells in the BM and spleen, which is associated with the hyper-responsive nature of NK cells to IL-15 (114). By directly interacting with JAK2, SOCS2 attenuated JAK2 activity and the corresponding JAK2–STAT5 signaling to negatively regulate NK cell differentiation (91). Increased NK cell differentiation has been observed in the absence of SOCS2 *in vivo* and *in vitro*, whereas the development advantage is reversed after the addition of a JAK2 inhibitor *in vitro*. In contrast to its effect on murine NK cells, SOCS2 has no influence on IL-15-mediated human NK cell differentiation *in vitro* but is essential for human NK cell effector function *via* the regulation of phosphorylated proline-rich tyrosine kinase 2 (Pyk2) (112). These discrepancies may be attributed to different protocols for mouse and human NK cell development *in vitro* or species differences. Although knockdown of SOCS3 in mice has no impact on NK cell development and maturation (90), a recent study revealed that SOCS3 suppressed IL-15-mediated STAT5 phosphorylation, correlating with the desensitization of NK cells to IL-15 simulation, resulting in disrupted NK cell terminal differentiation (115).

### IL-15–PI3K–AKT–mTOR Signaling for NK Cell Development

**Figure 6:**
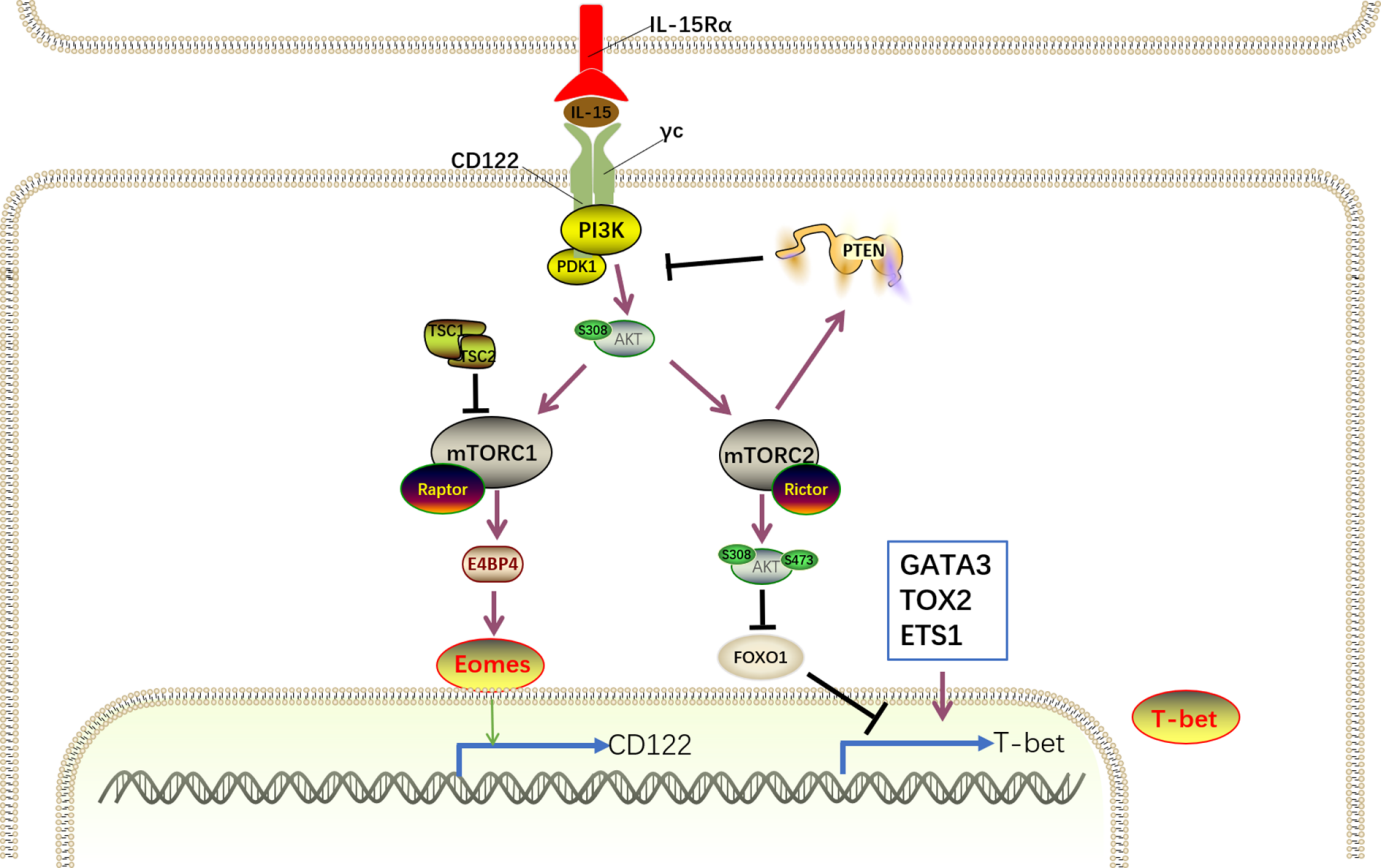
IL-15–PI3K–AKT-mTOR signaling for NK cell development. IL-15 complex interacts with its receptor IL-15R*β*/*γ*c on NK cells to trigger PI3K/AKT pathway autophosphorylation and activation and subsequent activation of mTORC1 and mTORC2. mTORC1 and mTORC2 differentially promote NK cell development in a cooperative and non-redundant manner primarily by divergent induction of corresponding transcription factor Eomes and T-bet. Eomes binds to the il2rb promoter and drives CD122 expression to maintain IL-15 responsiveness, generating a positive feedback loop to amplify the IL-15 signaling. Despite the negative regulation of T-bet expression by FoxO1, several transcription factors, including GATA3, TOX2, and ETS-1, promote T-bet expression. However, the activation of mTOR signaling is tightly modulated by cooperation of TSC1 and PTEN.

The interaction of IL-15 with its receptor on NK cells also activates the canonical downstream PI3K–AKT–mTOR pathway (**Figure 6**). Phosphoinositide 3-kinases (PI3Ks) are comprised of three subclasses, including class I, class II, and class III (116). The class I PI3Ks, which predominantly transduce signaling triggered by cytokine receptor, are heterodimeric enzymes that include a regulatory subunit (p85*α*, p50*α*, p55*α*, p85*β*, p55*γ*, and p101) and a catalytic subunit (p110*α*, p110*β*, p110γ, and p110*δ*). Mice exclusively or simultaneously lacking the PI3K subunits P110 *γ* and *δ* exhibit severely defective NK cell maturation and total numbers (116–119). Consistently, p110 *δ* mutations in patients impair the development and cytotoxic function of NK cells, leading to severe viremia, whereas rapamycin treatment partially rescues defective NK cells (117). Despite the multiple membranes of PI3Ks, it is unknown which subtypes are required for IL-15 signaling in NK cell development.

PI3K phosphorylates the three positions of the inositol ring of plasma membrane-associated phosphatidylinositol-4,5-bisphosphate [PI(4,5)P2] to generate PI(3,4,5)P3, which interacts with proteins containing pleckstrin homology (PH) domains, including the serine/threonine kinases phosphoinositide-dependent kinase (PDK1) and protein kinase B (PKB; also known as AKT), and localizes these proteins to membranes (120). The interaction between PI(3,4,5)P3 and AKT initiates conformational changes in AKT, allowing PDK1 to phosphorylate AKT at threonine 308 for AKT activation (121).

Subsequently, as an important downstream effector of PI3K/AKT signaling, the mammalian target of rapamycin (mTOR) is activated. mTOR, a serine/threonine protein kinase, includes two components, namely, mTOR complex1 (mTORC1) and mTORC2. Genetic studies have revealed that Raptor and Rictor are important components of mTORC1 and mTORC2, respectively, by defining their downstream substrates (122).

It was proposed that the activation of the IL-15R–PI3K–AKT–mTOR signaling cascade is dose-dependent. Specifically, low IL-15 concentrations only activate the phosphorylation of JAK/STAT5 signaling molecules, whereas the PI3K–AKT–mTOR pathway is further activated after exposure to high IL-15 concentrations (123). PI3K–AKT–mTOR signaling primarily regulates proliferation, differentiation, and maturation as well as NK cell effector function (124). The indispensable role of mTOR in controlling NK cell development was validated in mice with a specific deficiency in mTOR in NK cells in which NK cells almost disappeared in the peripheral organs, and the remaining NK cells in BM were severely blocked at the CD11b^−^CD27^+^ stage (123). Recent studies have demonstrated that mTORC1 and mTORC2 differentially promote NK cell development in a cooperative and non-redundant manner primarily by divergent induction of corresponding transcription factors, namely, T-bet and Eomes (33, 125). Intriguingly, mTORC1 and mTORC2 also positively or negatively regulate NK cell effector function, respectively. Ncr1^iCre^-mediated ablation of Raptor in mice results in disrupted mTORC1 function, leading to the impaired transition from CD27^+^CD11b^−^ to CD27^+^CD11b^+^ NK cells and reduced NK cell function. Conversely, terminal maturation from CD27^+^CD11b^+^ to CD27^−^CD11b^+^ NK cells is impeded in mice in the absence of Rictor, which is essential for mTORC2 metabolic signaling. However, Rictor-deficient NK cells display enhanced effector function.

E4 promoter-binding protein 4 (E4BP4), encoded by *Nfil3* (nuclear factor interleukin-3), is the predominant target downstream of mTORC1 (33, 126). Mechanistically, PDK1, a kinase downstream of PI3K, is thought to mediate IL-15-triggered mTORC1 and AKT phosphorylation to drive E4BP4 expression during NK cell development (127). Ectopic expression of E4BP4 rescued NK cell developmental defects in mTORC1-inactiavted and PDK1-deficient mice (126–128). Meanwhile, the absence of PDK1 in NK cells results in attenuated IL-15-triggered mTORC1 activation and significantly decreased E4BP4 expression (127). Moreover, the inactivation of mTORC1 diminishes the IL-15-mediated E4BP4 expression. These results suggest that the IL-15–PI3K–PDK1–mTORC1 signaling pathway is essential for E4BP4 induction. E4BP4 expression is initiated as early as the CLP stage and highly expressed in the iNK and mNK stages. *Nfil3−/–*mice display intact CLP compartment, and the population of NKPs, iNK cells, and mNK cells significantly reduced in the BM, indicating E4BP4 acts as early as CLP stage *via* an IL-15-independent manner and is essential for NK cell commitment. However, *Ncr1^iCre^*-mediated deletion of *Nfil3* has no effect on NK cell development (129), indicating that Nfil3 is dispensable for NK cell maturation, and other unknown signaling pathways compensate for the absence of Nfil3.

The induction of E4BP4 promotes the expression of Eomes, which binds to the il2rb promoter and drives CD122 expression to maintain IL-15 responsiveness (35). Mice with depletion of PDK1 or Eomes exhibit significant accumulation of CD27^+^CD11b^−^ NK cells but are devoid of terminally mature CD27^−^CD11b^+^ NK cells, and these findings resemble the findings in Raptor-deficient mice (33, 130, 131). Collectively, the IL-15–PI3K–PDK1–mTORC1–E4BP4–Eomes–CD122 pathway generates a positive feedback loop to amplify IL-15 signaling. However, mTORC1 activation is tightly modulated by Tuberous sclerosis 1 (Tsc1), which exhibits significantly increased expression after long-term IL-15 stimulation and forms a complex with Tsc2 with the aid of AKT (132).

Contrary to the indispensable role of mTORC1 in the early maturation of NK cells, mTORC2 is essential for the terminal maturation of NK cells from the CD27^+^CD11b^+^ to the CD27^−^CD11b^+^ stage. Previous studies have demonstrated that mTORC2 phosphorylates Akt at Serine 473 and augments its kinase activity, leading to the phosphorylation of FoxO1 by Akt (133, 134). Akt-triggered phosphorylation promotes modulator protein to interact with FoxO1, thereby inactivating it by blocking DNA binding and accelerating translocation from the nucleus to the cytosol (33, 135). This model has been further validated in NK cells through the discovery that mTORC2-inactivated NK cells display reduced phosphorylation of Akt^S473^ and FoxO1 (33). In addition, Ingenuity Pathway Analysis (IPA) found a remarkable enrichment of FoxO1 targets in mTORC2-inactivated NK cells. Furthermore, *in vitro* studies reported that IL-15 efficiently induces phosphorylation, and hence inactivation of FoxO1 in developing NK cells, together with the activation of mTOR signaling (136, 137). Based on these results, we speculate that FoxO1 is a direct target of the mTORC2–Akt^S473^ signaling axis that exists downstream of IL-15 signaling in NK cells. In contrast to the high expression of FoxO1 in NKp and iNKs, the level of FoxO1 is significantly decreased in mNKs (136). Apart from FoxO1, FoxO3 is also expressed by NK cells, although it is maintained at relatively low levels throughout NK cell development. Both *Ncr1-Cre -FoxO1^fl/fl^* and -*FoxO1^fl/fl^* mice exhibit accumulation of terminally differentiated CD27^−^CD11b^+^ NK cells, indicating FoxO1 and FoxO3 redundantly suppress NK cell maturation (137), contradicting the promoting effects of mTORC2. Owing to the weak expression of FoxO3, it is believed that FoxO1 plays a prominent role in NK cell development. However, in the same *Ncr1-Cre -FoxO1^fl/fl^* mice, Wang et al. reported a remarkable deficiency of iNK and mNK cells that is attributed to impaired FoxO1-mediated autophagy in iNK cells (136). Therefore, the distinct role of FoxO1 in NK cell development remains to be clearly clarified.

Several studies have demonstrated that the negative regulation of NK cell development by FoxO1 is associated with suppressed T-bet expression (33, 137). In humans, ChIP experiments showed that FoxO1 directly binds to the *Tbx21* promoter, promoting decreased T-bet expression. However, in mice, the recruitment of FoxO1 to the *Tbx21* proximal promoter region by Sp1, which is a FoxO1 protein binding partner, resulted in impaired transactivation of *Tbx21*, leading to disrupted T-bet expression. Consistently, the absence of FoxO1 in NK cells promotes T-bet mRNA and protein expression, whereas T-bet expression is decreased in NK cells with overexpression of FoxO1. In further support of this, Foxo1 and T-bet expression inversely correlate with each other during NK cell maturation. Immature NK cells express high levels of Foxo1, whereas T-bet is present in high amounts in terminally mature NK cells. Furthermore, in contrast to the accelerated maturation of NK cells in FoxO1^−/−^ mice, T-bet deficiency abrogated NK cell terminal maturation (40). Taken together, these results demonstrate that decreased levels of FoxO1 are necessary for NK cell maturation by releasing the negative regulation of T-bet.

Despite the negative regulation of T-bet expression by FoxO1, several transcription factors, including GATA binding protein 3 (GATA3), thymocyte selection-associated HMG box 2 (TOX2) and Ets proto-oncogene 1 (ETS-1), promote T-bet expression (138–141). Thus, inactivation of FoxO1 mediated by IL-15^−^PI3K^−^mTORC2 signaling coordinated with several transcription factors to promote T-bet expression. T-bet^−/−^ mice exhibited remarkably decreased NK cell populations in the periphery, but the NK cell number was modestly elevated in BM (142). This defect is attributed to the decreased expression of S1P5, which is induced by T-bet and responsible for NK cell egress from BM (143). In the absence of T-bet, NK cell maturation is specifically arrested at CD27^+^CD11b^+^stage, suggesting that T-bet is essential for NK cell terminal maturation (60). It has been proposed that T-bet promotes NK cell maturation by transiently inhibiting Eomes expression (36, 142). Consistent with this, T-bet levels are gradually increased during NK cell maturation, accompanied by the decreased expression of Eomes. T-bet also contributes to the induction of Zinc Finger E-box Binding Homeobox 2 (Zeb2) and B lymphocyte-induced maturation protein 1 (Blimp-1), which are critical for NK cell maturation (36, 40, 144). Thus, the IL-15R–PI3K–mTORC2–AKT–FoxO1–T-bet pathway determines the terminal maturation of NK cells.

In addition, mTORC2 suppresses mTORC1-mediated NK cell effector function by mainly downregulating SLC7A5 expression, which is downstream of STAT5 and regulates mTORC1 activity independent of AKT signaling (125, 145). Therefore, mTORC2 counteracts IL-15-mediated mTORC1 hyperactivation to prevent activation-induced NK cell apoptosis. Inversely, mTORC1 maintains IL-15–CD122–IL-15 signaling to sustain mTORC2 activity.

Phosphatase and tensin homolog (PTEN) directly antagonizes the PI3K–AKT pathway by specifically dephosphorylating PI(3,4,5)P3, which is downstream of PI3K and functions as an activator for downstream signaling proteins, including Vav, Akt, PDK1, and PI(4,5)P2 (146). Consistently, PTEN suppresses PI3K–AKT signaling and MAPK activation in humans, leading to compromised cytotoxic function (147). Conversely, the PTEN signaling pathway is impaired in Rictor-deficient NK cells with an inactive mTORC2 pathway, indicating that mTORC2 promotes PTEN expression to antagonize the PI(3,4,5)P3-mediated activation of mTORC2 (33, 148). Thus, a negative feedback exists between mTORC2 signaling and PTEN expression.

## Perspectives

NK cell development is tightly regulated by the interplay between intracellular transcription factors and extracellular signals, such as cognate ligands, chemokines, and cytokines. Notably, pleiotropic cytokine IL-15 is indispensable for the development of NK cells. Recently, immunotherapy has been applied in anti-cancer and anti-infection treatments. As an important component of immune cell, NK cells have potent cytotoxicity and cytokine production capacity, which allows effective eradication of malignant and infected cells in the absence of graft *versus* host disease (GVHD). Therefore, NK cells are promising for therapeutic utilization. The prerequisite of clinical application is to substantially expand mature NK cells *in vitro*. Correspondingly, understanding of the molecular mechanisms by which IL-15 promotes NK cell development and manipulation of IL-15 for proper NK cell expansion *in vitro* will improve NK cell-based therapeutic strategies (18).

NK cells are the earliest donor-derived lymphocytes recovering after HSCT, whose populations quickly reach donor levels within 1 month (149, 150). The well-established reconstitution of NK cells exhibits a protective effect against leukemia relapse and is associated with improved disease-free survival after HSCT (151–153). Although the level of IL-15 is remarkably high after HSCT, the immature CD56^bright^KIR^−^NK cells dominate the early reconstruction (149, 150). Investigating the role of IL-15 in NK cell development after HSCT contributes to better prognosis by intervening NK cell maturation.

Given the formidable efficacy in enhancing NK cell development, IL-15 is much more promising than other cytokines in controlling tumor progression and viral infections. Several murine immunotherapy trials have demonstrated that the administration of IL-15 efficiently drove the expansion and activation of NK cells and CD8+T cells *in vivo*, without stimulating the expansion of regulatory T cells which exert an immunosuppressive effect (154–156). However, due to its adverse effects, such as toxicities, hypotension, thrombocytopenia, IL-15 application was constrained (157). More basic research is required to optimize the structure of IL-15 before it can extended to clinical practice.

## Author Contributions

XW wrote the manuscript. X-YZ outlined the manuscript and made a deep intellectual contribution to the work. All authors contributed to the article and approved the submitted version.

## Funding

This study was supported by the National Key Research and Development Program of China (No. 2017YFA0104500), the National Natural Science Foundation of China (Nos. 81870140, 82070184), the Innovative Research Groups of the National Natural Science Foundation of China (No. 81621001), and Clinical Medicine Plus X - Young Scholars Project of Peking University (No. PKU2020LCXQ015) supported by “the Fundamental Research Funds for the Central Universities”, and Peking University People’s Hospital Research and Development Funds (No. RDX2019-14).

## Conflict of Interest

The authors declare that the research was conducted in the absence of any commercial or financial relationships that could be construed as a potential conflict of interest.
